# Physical environmental characteristics and individual interests as correlates of physical activity in Norwegian secondary schools: The health behaviour in school-aged children study

**DOI:** 10.1186/1479-5868-5-47

**Published:** 2008-09-29

**Authors:** Ellen Haug, Torbjørn Torsheim, Oddrun Samdal

**Affiliations:** 1 Research Centre for Health Promotion, Faculty of Psychology, University of Bergen, Christiesgt. 13, N-5020, Bergen, Norway

## Abstract

**Background:**

The school has been identified as a key arena for physical activity promotion for young people. Effective change of physical activity behaviour requires identification of consistent and modifiable correlates. The study explores students' interests in school physical activity and facilities in the school environment and examines their associations with students' participation in physical activity during recess and their cross-level interaction effect.

**Methods:**

This cross-sectional study was based on a national representative sample of Norwegian secondary schools and grade 8 students who participated in the Health Behaviour in School-aged Children (HBSC) 2005/06 study. The final sample comprised 68 schools and 1347 students. Physical environment characteristics were assessed through questionnaires completed by the principals, and students' physical activity and interests in physical activity were assessed through student self-completion questionnaires.

**Results:**

Most students were interested in more opportunities for physical activity in school. Multilevel logistic regression models demonstrated that students attending schools with many facilities had 4.49 times (95% Confidence Interval (CI) = 1.93–10.44) higher odds of being physically active compared to students in schools with fewer facilities when adjusting for socio-economic status, sex and interests in school physical activity. Also open fields (Odds Ratio (OR) = 4.31, 95% CI = 1.65–11.28), outdoor obstacle course (OR = 1.78, 95% CI = 1.32–2.40), playground equipment (OR = 1.73, 95% CI = 1.24–2.42) and room with cardio and weightlifting equipment (OR = 1.58, 95%CI = 1.18–2.10) were associated with increased participation in physical activity. Both students' overall interests and the physical facilitation of the school environment significantly contributed to the prediction of recess physical activity. The interaction term demonstrated that students' interests might moderate the effect of facilities on recess physical activity.

**Conclusion:**

The findings support the use of an ecological approach and multilevel analyses in the investigation of correlates of physical activity that allows for a broader understanding of the influence of and interaction between factors at multiple levels on physical activity behaviour. In the promotion of physical activity in lower secondary schools, the study suggests that programmes should include a focus on environmental facilitation and incorporate strategies to increase students' interests for school physical activity.

## Background

A considerable proportion of young people in the Western world fail to meet the 60 minutes of moderate to vigorous physical activity [1-4] recommended to gaining short- and long-term health benefits [5-8], with adolescents and girls reporting the lowest levels of physical activity [1-4]. A large number of national and cross-national policy plans across the western world have identified the school setting as a key arena for physical activity promotion for young people [9-13]. Numerous interventions have been undertaken to increase the amount of time spent in physical activity during school. However, the most recent reviews conclude that few programmes have documented substantial and sustainable effects [14-16]. To effectively change behaviour requires the identification of consistent and modifiable correlates of physical activity [17].

Much of the research on correlates of youth physical activity has focused on individual and sociocultural factors [18], but such correlates explain only some of the variance in physical activity behaviour [19]. As recognized by ecological theories [20], physical activity is a complex behaviour determined by a large number of influences at multiple levels. The principle behind this approach is that personal, socio-cultural, physical environmental and policy factors interact to promote or discourage participation in physical activity [21]. By drawing attention to the environmental influences on physical activity behaviour, ecological models open up a broader range of potential strategies to promote physical activity.

There are few evidence-based models for theorizing and testing the mechanisms underlying the interaction between specific environmental exposures and individual factors, and how these can influence physical activity behaviour [22]. In the *Youth Physical Activity Promotion (YPAP) *model, Welk [23] builds on existing research on developmental, psychological and behavioural characteristics specific to youth, and integrates the constructs of different theories [23]. The model divides the influential correlates of physical activity into three domains: (1) the individual-level *predisposing factors*, comprising the cognitive and affective considerations, represented by the two components "Is it worth it?" and "Am I able?"; (2) the *enabling factors *that include personal attributes (e.g., skills and fitness) and environmental or access variables; and (3) the *reinforcing factors *reflecting social influences. The environmental and reinforcing factors can directly influence physical activity levels because of their facilitating and stimulating effects on physical activity. According to Welk [23] there is a strong relationship between the components "Is it worth it?" and "Am I able?", because children would value what they are good at doing, and perceive that as worth doing, and likewise aim to become good at and pursue things they value.

Young people's interest in physical activity is an element of the construct "Is it worth it?" Interest has been found to be an important factor that drives children to adopt certain behaviours as a response to influences in the immediate environment. Individual interest is the psychological disposition of preferences for an activity or action that is based on the knowledge and the values that have been developed during an individual's interaction with this activity or action. Situational interest is the direct appealing effect of characteristics of an activity on a person. Both individual and situational interests are likely to have a combined impact on children decisions about what to do [24].

An implicit premise in the ecological approach is that determinants of physical activity behaviour are likely to be context specific [25]. It has been demonstrated that the relationships between specific psycho-social and environmental factors, and physical activity among youth vary according to the setting and type of physical activity [26]. The school arena offers several settings for physical activity, such as recess periods, including lunch breaks. The recess period can be defined as a regularly scheduled time for unstructured physical activity and play [27]. Such non-curricular periods can contribute up to 50% of the recommended 60 minutes of daily moderate to vigorous physical activity in students up to 12 years of age [28,29]. Norwegian secondary schools allocate, on average, a total of one hour of recess time each day [30]. The recess setting is therefore a potentially important time for increasing the uptake of physical activity by adolescents.

Secondary school students have identified greater accessibility to and availability of physical activity opportunities in school as important strategies for enhancing their participation in physical activity [31]. There is so far little empirical evidence of a relationship between the physical school environment and physical activity during recess [32]. Students are more likely to be physically active if they attend a school with high levels of sports equipment and fixed outdoor equipment along with supervision compared with students attending schools lacking this support [33]. Intervention studies show that providing extra sport equipment and supervision [34], extra sport materials along with computer-tailored individual follow-up [35], games equipment [28], and painted school playgrounds [36] can increase participation in physical activity during non-curricular school time. Only the latter intervention focusing on painted school playgrounds has demonstrated sustainable effects [37]. However, differences between school systems, infrastructure, environment, and social norms make it difficult to generalize findings between countries, and much of the research reported in the international literature has been conducted in the USA [16]. Research within the North European regions is therefore needed.

However, young people with access to environmental resources may not necessarily use them [23]. Kremers et al. recently suggested that individual-level factors other than demographic variables may moderate the environment-behaviour relationship [38]. During recess, students are free to do what they want. Whereas young children are thought to have a strong biological drive to be physically active [39], it could be hypothesized that the falling levels of physical activity observed through adolescent years [1-4] may be attributed to less interests in being physically active. A simultaneous examination of the physical school environment and students' interests in school physical activity as well as the unique and interacting effect of these factors in predicting physical activity during recess could give a better and more specific understanding of the complexity of physical activity behaviour within the school context.

Few of the earlier studies on environmental correlates of physical activity have accounted for the built-in multilevel structure when the samples have been recruited from school settings [32]. For example, the non-independence of students clustered within one school, because of students being influenced by shared and unique characteristics within that school or selection processes in enrolment that can give higher within-school correlations on the outcome variable, needs to be approached with multilevel modelling.

The aim of this Norwegian study was to extend previous research by: 1) explore secondary students' interests in physical activity in school and the availability of facilities in the school environment; 2) examine the relative strength of associations between students interests and physical activity during recess periods and between available facilities and physical activity during recess periods; and 3) examine the cross-level interaction of students' interests and facilities in predicting participation in recess physical activity.

## Methods

### Study sample and participants

The study was based on a nationally representative sample of Norwegian grade 8 students (13 years of age) participating in the Health Behaviour in School-Aged Children (HBSC) 2005/06 study. The HBSC study is a World Health Organization (WHO) Cross-National Survey in 11, 13 and 15 year olds that is conducted every fourth year and is currently carried out in 41 countries [40]. The original sample involving grade 8 students represented 115 schools and 2754 students. Of these, 79 schools (69%) with 1954 students enrolled in the sampled grade 8 classes took part in the study by completing a questionnaire. Eighty-two per cent (1595) of the students participated, and absence on the day that the survey was conducted was the most frequent cause of non-response. School-level data were also collected. Of the 79 participating schools, 11 did not return the school-level questionnaire. Students in these schools were excluded from the present study, and the final sample for the present study was 68 schools and 1347 students. Of these, 52.3% were boys and 47.7% were girls. The mean number of students enrolled in the sample schools was 301 (SD = 148; range, 9–712 students) with 43.3% of the students coming from urban and 56.7% from rural school areas. The mean number of grade 8 students participating in the sampled schools was 19.8 (SD = 6.8; range, 1–30). Most of the students (70.3%) were in the high socio-economic status (SES) group, 26.7% in the medium SES group, and 3% in the low SES group.

### Procedures

The data were collected in November-December 2005 in accordance with a standardized protocol [40]. A cluster sampling procedure was followed using school class as the sampling unit and with one participating class from each of the sampled schools. The school principal was asked to complete the school-level questionnaire and teachers received instructions on administering the student survey. Passive consent was received from parents or guardians. The student survey was carried out as a self-completion in-school questionnaire completed by students present during an ordinary class hour (45 minutes). Students were informed that their participation was voluntary and that responses would be treated anonymously. National ethical approval was obtained from the Regional Committee for Medical Research Ethics.

### Measurements

#### Physical activity

Physical activity during recess was measured with the item: "During recess, how OFTEN are you physically active in a way that makes you out of breath or makes you sweat?" with the following answer categories: "every recess", "not every recess but every day", "not every day but every week", "not every week", and "never". This variable was dichotomized with the first two response categories defined as "Daily physically active during recess". The wording of the item refers to vigorous physical activity [41,42]. However, the type of activity quantified by this item should not be interpreted only as vigorous physical activity because spontaneous behaviour by children and youth in non-organized physical activities characteristically involves alternating moderate to vigorous physical activity with short rest periods [42]. This item has been used in a previous HBSC survey in Norway [43]. A separate test-retest study of students aged 13 and 15 years indicated moderate stability for the item (intraclass correlation coefficient = 0.68) [44].

#### Interests in school physical activity

To assess their interests in school physical activity, students were asked to rate on a five-point Likert scale from (1) "strongly disagree" to (5) "strongly agree" how much they concurred with the following statements: "I would like various physical activities to be offered during recesses or lunch breaks", "I would like more physical education (PE) classes at school", "I am not interested in being more physically active during the school day", "I would like various physical activities to be offered after school" and "I want to have more school classes outdoors". The scores for the negatively worded item "I am not interested in being more physically active during the school day" were reversed. Cronbach's alpha coefficient for internal consistency for the items was 0.77. In the logistic regression model, the total scores were standardized with a score of 0 indicating the minimum and a score of 1 indicating the maximum total score.

#### Socio-economic status

The literature provides some support for effects of socio-economic factors on participation in physical activity [45] and sedentary [32,46] behaviour, and we included a measure of *SES*. SES was assessed using the Family Affluence Scale, which is a composite of four indicators: "Does your family have a car or a van?" ["No"(0), "Yes" (1), "Yes, two or more" (2)]; "Do you have your own bedroom?" ["No"(0), "Yes"(1)]; and "During the past year, how many times did you travel away on holiday (vacation) with your family?" ["Not at all" (0), "Once" (1), "Twice" (2), "More than twice" (3)]. "How many computers does your family own?" ["None" (0), "One" (1), "Two" (2), "More than two" (3)]. The two highest response categories ("2" and "3 or more") of the last two items (holidays and computers) were combined. The scores were added producing a scale that ranged from 0 (least affluent) to 7 (most affluent). An extensive description of the development and use of the scale has been given elsewhere [47]. For the descriptive analyses, a three-point ordinal scale was composed, using the following recoding of the scale: 0, 1, 2, or 3 = 1 (low); 4 or 5 = 2 (medium); and 6 or 7 = 3 (high).

#### School environment

For the international HBSC 2005/06 survey, a school-level questionnaire was developed through cross-national collaboration to examine the influence of the school environment on students' health behaviours [48].

#### Physical environmental characteristics

Physical environmental characteristics were assessed with the item: "Which facilities for physical activity exist in the indoor school area, the school yard (within 200 m), or in the school neighbourhood (200 to 2000 m)"? This item comprised a set of the following 16 natural or built characteristics: gymnasium or sport hall, swimming facilities, soccer fields, court space with permanent improvements for other ball games or activities, areas for skateboarding or skating, open field space with no markings, playground equipment, outdoor obstacle course, green fields or parks or nature reserve, wooded areas, water (sea, river or lake), ski tracks, ice-skating areas, fenced courts for ball games, climbing walls, and gym with cardio and weightlifting equipment. The five latter facilities were added to the Norwegian school-level questionnaire because they have been found to be present in Norwegian school settings [49]. Both the availability of (yes = 1 and no = 0) and the accessibility to (yes = 1 and no = 0) physical environmental characteristics in unstructured school time were assessed. Because availability and accessibility were highly correlated and the availability list contained less missing data, only data on the availability of facilities were used and a continuous variable labelled the "environment index" was generated.

### Data analysis

SPSS for Windows v. 14.0 was used for descriptive analyses. Chi-square tests were applied to examine sex differences in physical activity participation and in interests in school physical activity. Preliminary inspection of the school level data revealed that several of the variables had a considerable number of missing data. Since the multilevel analysis combines data from a school-level survey and a student-level survey, missing on one independent school level variable, would result in that all cases within that school would be excluded from the analysis. In cases where the school-level model only includes a few independent variables, this problem can be avoided with the multiple imputation (MI) procedure. Missing data are imputed based on all available data for the school information external to the model. Compared to other strategies for handling missing data, MI thus typically incorporates a richer set of information (including full information maximum likelihood (FIML)). The multiple imputations estimation requires the generation of multiple datasets, and performs data analysis on each of these data sets. Several software packages, including Mplus, provide automated analysis of and averaging across multiply imputed datasets. In the present study, multiple imputations were performed on the school-level data, using the software SOLAS 3.2. Five data sets were imputed using available school level information. These five datasets were merged with the individual-level student-survey, and prepared for analysis in Mplus. Two-level logistic regression was performed using the 'TWOLEVEL' command in Mplus. Adaptive quadrature with 15 quadrature points was used in the estimation.

A necessary requirement in multilevel modelling is that the dependent variable shows variation at multiple levels. The intraclass correlation (ICC) was computed using the formula presented in Snijders and Boskers (1999) [50]. If the ICC was sufficiently high, two sets of further analysis were planned. First, logistic regression was performed for each of the environmental factors at study. The objective of this analysis was to assess the relative strength of association with physical activity, adjusting for individual differences in interests. In a second more targeted set of analysis, hierarchical blockwise modelling of cross level main effects and cross-level interaction effects of environment was undertaken.

## Results

### Participation in physical activity

Overall, 41.5% of the boys and 32.6% of girls reported daily participation in physical activity during recess and school breaks, demonstrating significant sex differences.

### Interests in school physical activity

Table 1 depicts that most students strongly agreed or agreed with wanting more opportunities for physical activity during the school day. Sixteen percent strongly agreed or agreed with not being interested in being more physically active. For each statement, significant sex differences were observed; boys were more positive to additional opportunities for physical activities in various school contexts compared with girls.

**Table 1:** Grade 8 students' interests in and preferences for school physical activity

Interest variables	Gender	Strongly agree (%)	Agree (%)	Neither agree nor disagree (%)	Disagree (%)	Strongly disagree (%)
I would like various physical activities to be offered during recesses or lunch breaks	Boys**	28.3	30.2	29.2	5.6	6.7
	Girls	17.9	31.2	35.4	8.6	6.9
I would like more PE classes at school	Boys**	52.4	22.5	13.9	6.0	5.1
	Girls	31.2	23.9	22.1	14.1	8.8
I am not interested in being more physically active during the school day	Boys**	5.5	10.0	16.0	23.9	44.6
	Girls	5.1	11.3	23.9	29.1	30.7
I would like various physical activities to be offered after school	Boys*	26.8	26.7	29.8	8.6	8.0
	Girls	19.4	29.3	33.2	12.1	6.0
I want to have more school	Boys**	29.4	30.1	23.7	6.7	10.0
classes outdoors	Girls	16.1	24.4	15.1	15.1	11.5

*p < 0.05, **p < 0.001 between boys and girls, Pearson's chi-square test of significance.

### Environmental support for physical activity

A gym or sports hall; green fields, parks or nature reserve; soccer fields; and open field space with no markings were available in all or in almost all schools (table 2). Other frequently available facilities across schools were areas with improvements for other ball activities, wooded areas, and water (sea, river or lake). The intraclass correlation was 0.07, suggesting some variation in the level of activity between schools, and suggesting scope for multilevel modelling.

**Table 2:** Prevalence of environmental features available to the school sample

**Available facilities**	**%**
Gym or sports hall	100.0
Green fields, parks or nature reserve	98.5
Soccer fields	98.5
Open field space with no markings	97.0
Areas with improvements for other ball activities	91.2
Wooded areas	86.6
Water (sea, river, lake)	67.6
Swimming facilities	54.4
Ski track	54.4
Areas for skateboarding or skating	45.6
Room with cardio and weightlifting equipment	40.3
Ice-skating areas	38.8
Climbing walls	38.8
Fenced courtyard for ball games	33.8
Playground equipment	33.3
Outdoor obstacle course or activity trail	31.3

Table 3 shows the results of the multilevel logistic regression models for each of the environmental factors. Separate models were used to estimate the environment index and to evaluate the effect of each environmental factor after adjusting for compositional differences with regard to SES, sex and interests in school physical activity. The estimates are ordered in decreasing magnitude. The main effects model shows that student attending schools with many facilities had considerable higher odds of being physically active during recess periods on a daily basis compared with students in schools with fewer facilities. In addition, open fields, outdoor obstacle course, playground equipment and having a room with cardio and weightlifting equipment were also associated with participation in physical activity during recess. The other facilities did not yield any significant associations.

**Table 3:** Adjusted^a ^Odds Ratios (OR) from logistic regression models predicting daily participation in recess physical activity

**Available facilities**^#^	**OR**	**95% CI**		**Z**
Environment index	4.49*	1.93	10.44	3.48
Open field space with no marking	4.31*	1.65	11.28	2.98
Outdoor obstacle course or activity trail	1.78*	1.32	2.4	3.76
Playground equipment	1.73*	1.24	2.42	3.21
Room with cardio and weightlifting equipment	1.58*	1.18	2.1	3.10
Wooded areas	1.35	0.94	1.95	1.62
Ice-skating areas	1.29	0.91	1.83	1.45
Climbing walls	1.25	0.9	1.74	1.32
Swimming facilities	1.17	0.84	1.63	0.94
Water (sea, river, lake),	1.07	0.75	1.52	0.38
Areas for boarding/skating	1	0.72	1.38	-0.01
Ski track	1	0.72	1.38	-0.02
Soccer fields	0.9	0.76	1.07	-1.17
Areas with improvements for other ball activities	0.79	0.58	1.09	-1.41
Fenced courtyard for ball games	0.74	0.49	1.11	-1.44

^**a **^Adjusted for SES, sex and individual interests in school physical activity.

^# ^Analyses were not conducted for 'gym or sports hall' and 'green fields, parks or nature reserve' because there were no variance in these variables on the student level.

* *p *< 0.01.

CI, confidence interval.

**Figure 1:**
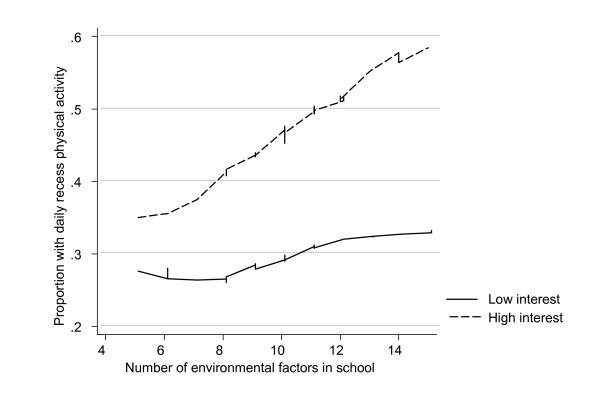
The interaction of environment by interests.

Table 4 shows the results of multilevel logistic regression models using hierarchical blockwise modelling. Three models were tested. The first model included individual factors only. In the second model, main effects of the contextual variable were added to examine the cross-level main effects of environmental factors on individual daily recess activity. Finally, in the third model, cross-level interaction effects were included to examine whether the impact of individual factors interacted with contextual factors in the prediction of daily recess activity. The main effects model indicated that, after controlling for gender and SES, the interests' index was a statistically significant predictor of daily recess physical activity. This was demonstrated by a change in physical activity of 2.36 logits from lowest to highest interest scores. The cross-level main effect of the environment index showed a change in daily recess physical activity of 1.5 logits from an impoverished to an enriched environment. The environmental index accounted for a substantial part of the random variation in physical activity during recess, indicated by a drop from 0.16 to 0.11. The interaction term between the environment and interests in the third model was statistically significant as indicated by the Wald test (Z = 2.46), but the overall block was not statistically significant under Likelihood Ratio test (deviance = 1525.03-1521.32 = 3.71, p < 0.108, two-tailed test). The strong positive regression weight (4.32 logits) for the interaction term indicated that the association between the environment and physical activity was stronger for students with high interests, as displayed in figure 1. To check for confounding factors, interaction terms for environment by SES and by gender were included in the model, but these terms did not reach statistical significance, and did not change the magnitude of interaction between environment and interests.

**Table 4:** Regression weights, standard errors (SE), and Z-scores from multilevel logistic regression models predicting daily physical activity during recess.

	**Individual**			**Contextual**			**Crosslevel interaction**		
	B	SE	Z	B	SE	Z	B	SE	Z

Intercept	-0.52	0.09	-5.89***	-0.55	0.08	-7.28***	-0.55	0.08	-7.29***
SES	-0.37	0.373	-0.99	-0.39	0.37	-1.05	-0.40	0.37	-1.08
Gender	-0.13	0.144	-0.91	-0.12	0.14	-0.82	-0.11	0.14	-0.79
Interests index	2.36	0.33	7.18***	2.33	0.33	7.13***	2.34	0.31	7.45***
Environmental index				1.50	0.43	3.48***	1.42	0.43	3.30***
Environment by interests							4.32	1.76	2.46*
Random effect	0.16	0.07	2.21	0.11	0.07	1.56	0.11	0.07	1.58

Deviance	1532.80			1525.03 (0.37)			1521.32 (0.34)		

* p < 0.05, *** p < 0.001

Figure 1 shows a loess-smoothed curve of recess activity on number of environmental factors for students with low and high interests, respectively. With few facilities available, the proportion of physically active students was low in both interest groups. With more facilities available, the differences in the proportion being active between the interest groups increased.

## Discussion

This is to our knowledge the first study to examine simultaneously the effects of students' interests in physical activity, environmental factors, and their interaction effect on participation on school physical activity. The results demonstrate that environmental support was a unique contributor to daily physical activity after controlling for all individual level variables, including students' interests, and accounted for a substantial part of the random variation across schools. The odds ratio of being physically active was 4.5 times higher in schools with many facilities for physical activity than in schools with fewer. The findings are in line with previous studies, demonstrating that physical environmental characteristics in the school setting have the ability to influence students' activity level [28,33,34,51]. The results also support the observation that the students identify their own strategies to increase their participation in school physical activity [31]. From a public health perspective, these findings are encouraging, because environmental changes can influence the whole student population.

However, our data extend previous research by indicating that students' interests in school physical activity may moderate the impact of facilities on participation in physical activity during recess. As displayed in figure 1, the differences in participation rate were small in schools deprived of environmental support, and generally low in both interest groups. With more facilities available, a strong increase in the proportion of students being active in the 'high interest group' was seen, but no noteworthy changes in the group with weak interests. The interaction term was statistically significant as indicated by the Wald test. However, there was only a trend towards significance observed for the ML-test, demonstrating uncertainties in the estimates. The latter may be due to (1) the multiple imputation procedure that deliberately incorporates random variations in the estimates, and (2) the relatively small number of schools that were included, and an inaccurate measure of physical activity, which may result in a lack of statistical power. The study finding is therefore inconclusive and further research is required to obtain a more in-depth insight into the impact of interests and environmental facilities on physical activity. If additional research would support the findings of the present study, a focus on developing stronger interests in school physical activity would be essential. Nevertheless, the findings are consistent with the YPAP model suggesting that physical environmental support is an enabling factor that is necessary but not sufficient for physical activity behaviour [23].

We observed no clear threshold in the optimal level of environmental support needed to generate maximum participation in physical activity for motivated students, and it is uncertain whether more facilities would engage a larger proportion of this student group in physical activity during recess. The increased likelihood of being active in schools with richly facilitated environments can be attributed to several factors. Sufficient space and a selection of facilities and settings where one can be physically active may give students more opportunities and lessen the competition between students for the spaces. The number of facilities could also reflect the size of the available outdoor area at school. In a previous study, both school campus area and play area per student were positively associated with physical activity measured with an accelerometer, but it was not possible to detect whether the increase was attributed to more transport related walking or recreational activities [52]. In our study, after adjusting for individual level factors, characteristics such as "fields with no markings", "outdoor obstacle course", "playground equipment", and "gym with equipment for cardio and weight training" were all associated with higher odds for being physically active. However, these bivariate predictors should be interpreted with cautiousness. We note that some of the facilities in the schools could have been used for purposes other than the activity for which they were labelled specifically. Furthermore, the facility "playground equipment" could be an indicator of a school that also has younger children enrolled, which could influence the social climate for physical activity, as younger children tend to be naturally more active than older ones. Future research should include comprehensive observational studies to get a better understanding of students' use of the facilities and their preferences.

In the study, students' overall interests were a strong predictor for physical activity. It is likely that students' interests in school physical activity reflect a set of considerations in this specific setting, including the various elements of the "Is it worth it?" and the related "Am I able?" component [53]. A review by Rees et al. [54] showed that most adolescents have positive beliefs about physical activity but that a number of barriers prevent young people from being active. It is thought that when perceived barriers outweigh perceived benefits, a person would be less predisposed to participate in physical activity [55], even if students have many environmental characteristics available.

Most of the students were interested in more opportunities for physical activity in the various contexts of the school setting. However, girls were significantly less interested and also less likely to participate in daily physical activity compared with boys. Especially girls experience many barriers to participate in physical activity such as not feeling competent enough, negative reactions from peers about skills, self-consciousness about one's body, lack of time and lack of relevant facilities [54,56]. A dislike of competitive activities, highly structured activities, or those organized by others have often been reported as reasons for not participating in physical activity, particularly among adolescent girls [54]. These factors may result in girls being less interested in physical activity. It has been found that during recess, the fields are occupied mainly by boys for soccer and football [57,58], and the unstructured format of open gym time allows boys to dominate the space [59]. Although facilities and areas are physically available for boys and girls, not all girls may perceive these as accessible or as enjoyable as boys. In studies that have examined students' opinions of promising approaches for increasing their physical activity levels, young women wanted more equal opportunities and more choices of activities in school and programmes, including activities such as dancing and gymnastics [31,54]. However, in the present study the interaction term environment by gender did not reach statistical significance, and did not change the magnitude of interaction between environment and interests, which suggests that a richly facilitated environment had an effect on both genders. This information is encouraging, and demonstrates that the school environment is a promising arena for promoting physical activity among adolescents.

Physical activity participation during recess did not differ significantly between SES groups, and SES did not confound the relationship between the environment index and physical activity participation. However, only 3% of the sample was in the low SES group, which reduced the variance of this variable. Nevertheless, the results are encouraging and support the idea that the school setting is an arena to reduce social inequalities in health, in this case, participation in physical activity.

### Limitations

We acknowledge some limitations related to the use of self-report. One important limitation is the use of subjective assessment of physical activity. Only moderate correlations have been found between self-reports and more objective measurements among children [60,61]. However, in our study, the physical activity item referred to a specific setting and level of intensity, which could have increased the accuracy of reporting. In addition, dichotomizing the responses might have increased the number of students categorized correctly. The use of self-reported assessment of environmental factors may also have influenced the results. We do not know whether the principals interpreted each area in the same way or how much they knew about the characteristics in the school environment. Objective monitoring or observation of the environment could have strengthened the validity of the environmental measures and should be considered in future studies.

## Conclusion and implications

Physical environmental factors and students' interests in school physical activity were significant predictors of daily participation in physical activity during recess. The results suggest that students' interests may moderate the effect of environmental facilities, with strong associations between the physical activity and the environment found only for students that had strong interests in school physical activity. The findings support the use of an ecological approach in the investigation of correlates of physical activity that allows for a broader understanding of the complex mechanisms of individual and environmental correlates involved in shaping physical activity behaviour.

Therefore, in the promotion of physical activity in lower secondary schools, the present study suggests that programmes should incorporate strategies to increase students' interests and motivation for school physical activity as well as having a specific focus on environmental facilitation. Building on the experiences from the large number of earlier interventions that have targeted students directly would be important in the development of the individual level intervention components. The impact of social factors was not addressed in the present study but needs further investigation. Several studies have found that social support from adults and peers is an important factor for physical activity uptake and maintenance [62], this has also been demonstrated for physical activity uptake during non-curricular school time [26].

Recent literature reviews have concluded that whole-school interventions that address individual, social and environmental factors are more promising approaches to increasing physical activity among schoolchildren compared with curriculum-only programmes [14-16].

Building on the students' own ideas about what should be included in a physical activity-friendly school setting seems highly relevant, and could indirectly influence their involvement in physical activity through stronger interests as well as enjoyment. Because school interventions target the entire student population and new student population groups will be exposed to the school environment over time, such interventions can increase the impact of changes at the school level. Exposing students to a variety of physical activity opportunities in the school environment can also be a strategy to introduce children to physical activities they can perform during their leisure time, which subsequently should raise their total levels of daily physical activity. Similarly, improving students' interests in physical activity in the school setting may also influence their motivation for physical activity outside school hours. The potential effect of a comprehensive approach might therefore not necessarily be limited to the school setting.

## Competing interests

The authors declare that they have no competing interests.

## Authors' contributions

EH contributed to the design, the interpretation, the drafting and the coordination of the manuscript. TT advised on the design, performed the statistical analyses and contributed in the interpretation of the data. OS advised on the design, provided input at each stage of the manuscript draft and read the paper critically for theoretical content and interpretation of findings. All authors read and approved the final manuscript.
